# Familial Clusters of Coronavirus Disease in 10 Prefectures, Japan, February−May 2020

**DOI:** 10.3201/eid2703.203882

**Published:** 2021-03

**Authors:** Reiko Miyahara, Naho Tsuchiya, Ikkoh Yasuda, Yura K. Ko, Yuki Furuse, Eiichiro Sando, Shohei Nagata, Tadatsugu Imamura, Mayuko Saito, Konosuke Morimoto, Takeaki Imamura, Yugo Shobugawa, Hiroshi Nishiura, Motoi Suzuki, Hitoshi Oshitani

**Affiliations:** National Center for Global Health and Medicine, Tokyo, Japan (R. Miyahara);; National Institute of Infectious Diseases, Tokyo (R. Miyahara, M. Suzuki);; Tohoku University, Miyagi, Japan (N. Tsuchiya, Y.K. Ko, S. Nagata, T. Imamura, M. Saito, H. Oshitani);; Nagasaki University, Nagasaki, Japan (I. Yasuda, E. Sando, K. Morimoto); Kyoto University, Kyoto, Japan (Y. Furuse, H. Nishiura);; Japan International Cooperation Agency, Tokyo (T. Imamura);; National Center for Child Health and Development, Tokyo (T. Imamura);; Niigata University Graduate School of Medical and Dental Sciences, Niigata, Japan (Y. Shobugawa);; Hokkaido University, Hokkaido, Japan (H. Nishiura)

**Keywords:** coronavirus disease, COVID-19, respiratory infections, familial clusters, prefectures, Japan, severe acute respiratory syndrome coronavirus 2, SARS-CoV-2, viruses

## Abstract

The overall coronavirus disease secondary attack rate (SAR) in family members was 19.0% in 10 prefectures of Japan during February 22–May 31, 2020. The SAR was lower for primary cases diagnosed early, within 2 days after symptom onset. The SAR of asymptomatic primary cases was 11.8%.

As of May 31, 2020, Japan had reported >16,800 confirmed coronavirus disease (COVID-19) cases and 890 related deaths. The cluster-based approach is one of the pillars of control measures in Japan (*1*). Sixty-one clusters were documented in healthcare facilities, restaurants, workplaces, and music venues during January–April 2020 (*2*). However, the transmission within households, one of the highest-risk settings, has not been fully investigated.

A meta-analysis of 43 studies showed that the pooled household secondary attack rate (SAR) was 18.1%, and heterogeneity ranged from 3.9% to 54.9% (*3*). Heterogeneity of SAR could occur because of variations in susceptibility to infection (*3*), variations in exposure (*4*), and variations in infectiousness. The primary cases of infectiousness defined by age, sex, and symptoms were less studied in the different settings. Furthermore, there were few reports of SAR among asymptomatic primary cases (*3*,*5*,*6*). Therefore, we estimated the SAR of COVID-19 and assessed the effects of age and sex of primary cases, symptoms of primary cases, and the time between diagnosis and symptom onset for primary cases on infectiousness in familial clusters.

## The Study

Among 47 prefectures in Japan, 10 prefectures (Aomori, Akita, Gunma, Tochigi, Toyama, Shiga, Okayama, Kochi, Saga, and Kagoshima) (Appendix Figure 1) that showed a relatively low COVID-19 prevalence posted laboratory-confirmed cases and contact-tracing results on their websites (Appendix Table 1). In this study, we collected basic characteristics of cases from the reports issued during February 22–May 31, 2020, on those websites. The websites did not provide characteristics of uninfected close contacts or details of residence of family members. During the study period in Japan, doctors provided diagnoses of COVID-19 by using real-time PCR and reported cases to healthcare centers. These centers listed close contacts according to whether they spent >15 min in face-to-face contact and conducted follow-up by telephone for >14 days to monitor their symptoms.

Persons who had any COVID-19–related signs/symptoms, such as fever, cough, and fatigue, were categorized as having symptomatic cases. Asymptomatic cases were those without any symptoms at diagnosis. During this study period, all confirmed case-patients were hospitalized after they were given a diagnosis. Suspected case-patients and asymptomatic close contacts self-quarantined at home. Healthcare centers in 8 prefectures performed PCRs for close contacts regardless of their symptoms. One prefecture did not show the strategy of PCR testing for asymptomatic contacts, and 1 prefecture performed PCRs for symptomatic contacts, such as persons who had fever and respiratory symptoms.

In this study, we defined a primary case as the first case to show development of symptoms and to be diagnosed in a family or the first diagnosed asymptomatic case in a family who had an apparent history of contact with a nonfamilial COVID-19 case-patient. We defined secondary cases as laboratory-confirmed cases from the list of close family contacts of primary case-patients. Because websites provided only symptoms at diagnosis, we could not identify presymptomatic cases. We calculated SAR as the proportion of secondary cases of family close contacts among the total number of family close contacts and determined the SAR, risk ratio, and 95% CI, stratified by the characteristics of the primary case-patients. We compared the SAR before and after the declaration of the state of emergency on April 16. All statistical analyses were conducted by using Stata version 14.0 (StataCorp, https://www.stata.com).

During February 22–May 31, 2020, the 10 prefectures reported 306 primary cases and 775 family close contacts from 306 families. Eighty-seven primary cases were associated with 147 family secondary cases (Table 1; Appendix Figure 2). The overall SAR was 19.0%. Among 28 asymptomatic primary cases, 7 caused family clusters (Table 2; Appendix Table 2), and the SAR was 11.8%. Eight prefectures that tested for asymptomatic contacts showed an SAR that was 1.77 times higher than the SAR for 2 prefectures that used a nontesting strategy. The age-stratified SAR was higher for persons 60–69 years of age (36.5%) and persons <20 years of age (23.8%) than for persons 20–29 years of age (13.3%), persons 30–39 years of age (20.4%), persons 40–49 years of age (10.1%), and persons 50–59 years of age (16.1%) (Table 2).

**Table 1:** Characteristics of primary and secondary case-patients in households of familial clusters of coronavirus disease in 10 prefectures, Japan, February−May, 2020*

Characteristic	Primary	Secondary
No. case-patients	306	147
Sex		
F	152 (49.7)	82 (55.8)
M	153 (50.0)	64 (43.5)
Unknown	1 (0.3)	1 (0.7)
Age, y		
0–19	11 (3.5)	28 (19.0)
20–29	48 (15.7)	14 (9.5)
30–39	36 (11.8)	16 (10.9)
40–49	58 (19.0)	8 (5.4)
50–59	57 (18.6)	24 (16.3)
60–69	43 (14.1)	29 (19.7)
70–79	31 (10.1)	14 (9.5)
>80	22 (7.2)	14 (9.5)
Unknown	0 (0)	1 (0.7)
Contact history to COVID-19 nonfamilial cases		
No	146 (47.7)	147 (100)
Yes	159 (52)	0
Unknown	1 (0.3)	0
Symptom		
Symptomatic	271 (88.6)	103 (70.1)
Asymptomatic	28 (9.2)	39 (26.5)
Unknown	7 (2.3)	5 (3.4)
Median time from symptom onset to diagnosis, d (IQR)	6 (4–9)	5 (2.5–9)
Confirmed date of primary case		
On or before April 16	179 (58.5)	NA
After April 16	127 (41.5)	NA
Policy of testing for asymptomatic contacts		
No testing, 2 prefectures	54 (17.6)	16 (10.9)
Testing for asymptomatic contacts, 8 prefectures	252 (82.4)	131 (89.1)
*Values are no. (%) unless otherwise indicated. COVID-19, coronavirus disease; IQR, interquartile range; NA, not applicable.		

**Table 2:** Characteristics of primary cases in households and SAR categorized for households of familial clusters of coronavirus disease in 10 prefectures, Japan, February−May, 2020*

Variable	No. (%) primary cases	No. family contacts	No. secondary infected cases	No. symptomatic secondary infected cases	No. asymptomatic secondary infected cases	SAR, % (95% CI)	Risk ratio (95% CI)
Overall	306 (100)	775	147	103	39	19.0 (16.3–21.9)	
Sex							
F	152 (49.7)	408	68	50	14	16.7 (13.2–20.6)	Referent
M	153 (50.0)	366	79	53	25	21.6 (17.5–26.2)	1.29 (0.97–1.73)
Unknown	1 (0.3)	1	1	0	1	NA	NA
Age, y							
<1–19	10 (3.6)	42	10	7	3	23.8 (12.1–39.5)	Referent
20–29	48 (15.7)	135	18	15	2	13.3 (8.1–20.3)	0.56 (0.28–1.12)
30–39	36 (11.8)	103	21	15	6	20.4 (13.1–29.5)	0.85 (0.44–1.66)
40–49	58 (19.0)	139	14	9	5	10.1 (5.6–16.3)	0.42 (0.20–0.88)
50–59	57 (18.6)	155	25	13	9	16.1 (10.7–22.9)	0.68 (0.35–1.30)
60–69	43 (14.1)	85	31	20	10	36.5 (26.3–47.6)	1.53 (0.83–2.81)
70–79	31 (10.1)	53	11	10	1	20.8 (10.8–34.1)	0.87 (0.41–1.85)
>80	22 (7.2)	63	17	14	3	29.4 (23.2–36.2)	1.13 (0.58–2.23)
Contact history with nonfamilial COVID-19 cases							
No	146 (47.7)	357	91	64	24	25.4 (21.0–30.3)	1.90 (1.4–2.57)
Yes	159 (52.0)	417	56	39	15	13.4 (10.3–17.1)	Referent
Unknown	1 (0.3)	1	0	0	0	NA	NA
No. household contacts per primary case							
1	88 (28.8)	88	17	15	2	19.3 (11.7–29.1)	Referent
2	75 (24.5)	150	26	16	8	17.3 (11.6–24.4)	0.90 (0.52–1.56)
3	82 (26.8)	246	47	32	13	19.1 (14.4–24.6)	0.90 (0.60–1.63)
4	35 (11.4)	140	36	27	8	25.7 (18.7–33.8)	1.33 (0.80–2.22)
>5	26 (8.5)	151	21	13	8	13.9 (8.8–20.5)	0.72 (0.40–1.29)
Symptoms							
Symptomatic	271 (88.6)	661	136	98	33	20.6 (17.6–23.9)	Referent
Asymptomatic	28 (9.2)	93	11	5	6	11.8 (6.1–20.2)	0.57 (0.32–1.02)
Unknown	7 (3.6)	21	0	0	0	NA	NA
Time from symptom onset to diagnosis, d, n = 271							
0–2	30 (11.1)	65	4	3	1	11.6 (5.1–21.6)	Referent
3–7	130 (48.0)	319	63	42	21	19.8 (15.5–24.5)	3.21 (1.21–8.51)
8–14	94 (34.7)	230	51	40	8	22.2 (17.0–28.1)	3.60 (1.35–9.6)
>14	17 (6.3)	45	18	9	9	40.0 (25.7–55.7)	6.50 (2.36–17.93)
Confirmed date of primary case							
Feb 22–Apr 16	179 (58.5)	448	78	57	16	17.4 (14.0–21.3)	Referent
Apr 17–May 31	127 (41.5)	328	69	46	23	21.0 (16.8–25.9)	1.21 (0.90–1.61)
Policy of testing for asymptomatic contacts							
No testing, 2 prefectures	54 (17.6)	138	16	12	1	11.6 (6.8–18.1)	Referent
Testing for asymptomatic contacts, 8 prefectures	252 (82.4)	637	131	91	38	20.6 (17.5–23.9)	1.77 (1.09–2.88)

*COVID-19, coronavirus disease; NA, not applicable; SAR, secondary attack rate.

With increasing time from symptom onset to diagnosis, the SARs in households increased from 11.6% (>2 days) to 40.0% (>14 days) (Table 2). When the data were stratified for analysis by the number of household contacts, 4 household contacts showed the highest SAR (25.7%). After a quarantine at home was requested from the government on April 16, the SAR increased from 17.4% to 21.0%, but the risk ratio did not reach statistical significance.

## Conclusions

This family cluster analysis in the 10 prefectures of Japan showed that the overall SAR of the family cluster was estimated to be 19.0% in Japan. Meta-analysis from 43 household transmission studies estimated a SAR of 18.1% (*3*): 3.9% in Singapore (*7*), 4.6% in Taiwan (*8*), 10.3%–54.9% in China (*9*–*12*), and ≈30% in the United States (*13*) and Norway (*14*). In addition, the SAR of asymptomatic primary cases was 11.8% in our study, which was higher than the 0%–4.4% reported in a limited number of previous studies (*6*,*15*). The SAR heterogeneity might have been dependent on the surveillance protocol for asymptomatic contacts. The studies in the United States (*13*) and Norway (*14*), which had high SARs, detected secondary cases by using serologic tests. Our study also indicated that 8 prefectures that tested for asymptomatic contacts showed a 1.8 times higher SAR than did 2 prefectures that tested only for symptomatic contacts. A low proportion of diagnoses of asymptomatic cases might underestimate the SAR.

We showed that SAR was higher for persons <1–19 years of age and >60 years of age than for other age groups. High infectivity for the younger age group (*6*) and the older age group (*4*) was reported from South Korea and China, as in our study, but most other studies did not show significant differences in SAR by age of primary case-patients (*9*,*13*). Age-dependent infectivity might be associated with household lifestyles, family structure, and clinical conditions (*9*). Meta-analysis showed that the sex of the primary case-patient was not associated with transmission (*5*).

If primary cases were detected <2 days of symptom onset, the SAR was lower than that for primary cases detected >2 days after symptom onset. This finding was related to the low SAR for case-patients who had a contact history because they could receive PCRs, as close contacts did earlier, and might have had a short time of exposure to family members. Our results were concordant with previous studies showing an increased risk for transmission as the contact duration was prolonged (*4*), as well as the effect of quarantining index case-patients when symptoms were reported (*10*).

The first limitation of our study is that symptomatic cases diagnosed during the presymptomatic period might have been classified as asymptomatic cases. Second, the number of asymptomatic cases might have been underreported because of different testing protocols among prefectures. Third, we might have misclassified the primary cases if a coprimary case existed or the direction of transmission between asymptomatic cases and symptomatic cases was not clear.

In summary, our study results provide us with useful implications of the high SAR of asymptomatic primary case-patients and contacts with long exposure times to primary case-patients. Self-quarantine and rapid isolation of confirmed case-patients from households after symptom onset might be needed to reduce transmission in families.

Appendix Additional information on familial clusters of coronavirus disease in 10 prefectures, Japan, February−May, 2020.

## References

[1] Oshitani H. Experts Members of The National COVID-19 Cluster Taskforce at Ministry of Health, Labour and Welfare, Japan. Cluster-based approach to coronavirus disease 2019 (COVID-19) response in Japan, from February to April 2020. Jpn J Infect Dis. 2020;73:491–3. 10.7883/yoken.JJID.2020.363 32611985

[2] Furuse Y, Sando E, Tsuchiya N, Miyahara R, Yasuda I, Ko YK, et al. Clusters of coronavirus disease in communities, Japan, January–April 2020. Emerg Infect Dis. 2020;26:2176–9. 10.3201/eid2609.202272 32521222 PMC7454082

[3] Koh WC, Naing L, Chaw L, Rosledzana MA, Alikhan MF, Jamaludin SA, et al. What do we know about SARS-CoV-2 transmission? A systematic review and meta-analysis of the secondary attack rate and associated risk factors. PLoS One. 2020;15:e0240205. 10.1371/journal.pone.0240205 33031427 PMC7544065

[4] Xin H, Jiang F, Xue A, Liang J, Zhang J, Yang F, et al. Risk factors associated with occurrence of COVID-19 among household persons exposed to patients with confirmed COVID-19 in Qingdao Municipal, China. Transbound Emerg Dis. 2020;Jul 20 [Epub ahead of print]. 10.1111/tbed.13743 PMC7404761 32688447

[5] Madewell ZJ, Yang Y, Longini IM Jr, Halloran ME, Dean NE. Household transmission of SARS-CoV-2: a systematic review and meta-analysis. JAMA Netw Open. 2020;3:e2031756. 10.1001/jamanetworkopen.2020.31756 33315116 PMC7737089

[6] Park SY, Kim Y-M, Yi S, Lee S, Na B-J, Kim CB, et al. Coronavirus disease outbreak in call center, South Korea. Emerg Infect Dis. 2020;26:1666–70. 10.3201/eid2608.201274 32324530 PMC7392450

[7] Pung R, Park M, Cook AR, Lee VJ. Age-related risk of household transmission of COVID-19 in Singapore. Influenza Other Respir Viruses. 2020;•••:irv.12809. 10.1111/irv.12809 32990399 PMC7646651

[8] Cheng HY, Jian SW, Liu DP, Ng TC, Huang WT, Lin HH; Taiwan COVID-19 Outbreak Investigation Team. Contact tracing assessment of COVID-19 transmission dynamics in Taiwan and risk at different exposure periods before and after symptom onset. JAMA Intern Med. 2020;180:1156–63. 10.1001/jamainternmed.2020.2020 32356867 PMC7195694

[9] Jing Q-L, Liu M-J, Zhang Z-B, Fang L-Q, Yuan J, Zhang A-R, et al. Household secondary attack rate of COVID-19 and associated determinants in Guangzhou, China: a retrospective cohort study. Lancet Infect Dis. 2020;20:1141–50. 10.1016/S1473-3099(20)30471-0 32562601 PMC7529929

[10] Li W, Zhang B, Lu J, Liu S, Chang Z, Peng C, et al. The characteristics of household transmission of COVID-19. Clin Infect Dis. 2020;71:1943–6. 10.1093/cid/ciaa450 32301964 PMC7184465

[11] Luo L, Liu D, Liao X, Wu X, Jing Q, Zheng J, et al. Contact settings and risk for transmission in 3,410 close contacts of patients with COVID-19 in Guangzhou, China: a prospective cohort study. Ann Intern Med. 2020;173:879–87. 10.7326/M20-2671 32790510 PMC7506769

[12] Zhang J, Litvinova M, Liang Y, Wang Y, Wang W, Zhao S, et al. Changes in contact patterns shape the dynamics of the COVID-19 outbreak in China. Science. 2020;368:1481–6. 10.1126/science.abb8001 32350060 PMC7199529

[13] Lewis NM, Chu VT, Ye D, Conners EE, Gharpure R, Laws RL, et al. Household transmission of SARS-CoV-2 in the United States. Clin Infect Dis. 2020;•••:ciaa1166. 10.1093/cid/ciaa1166 33185244 PMC7454394

[14] Cox RJ, Brokstad KA, Krammer F, Langeland N, Blomberg B, Kuwelker K, et al.; Bergen COVID-19 Research Group. Seroconversion in household members of COVID-19 outpatients. Lancet Infect Dis. 2020;S1473-3099(20)30466-7. 10.1016/S1473-3099(20)30466-7 PMC7295487 32553187

[15] Chaw L, Koh WC, Jamaludin SA, Naing L, Alikhan MF, Wong J. Analysis of SARS-CoV-2 transmission in different settings, Brunei. Emerg Infect Dis. 2020;26:2598–606. 10.3201/eid2611.202263 33035448 PMC7588541

