# Supplementary material for: Familial Clusters of Coronavirus Disease in 10 Prefectures, Japan, February−May 2020
Source: Emerg Infect Dis. 2021 Mar;27(3):915–8. doi: 10.3201/eid2703.203882 (PMC7920650; doi:10.3201/eid2703.203882)
Supplement: Appendix — Additional information on familial clusters of coronavirus disease in 10 prefectures, Japan, February−May, 2020. [file 20-3882-Techapp-s1.pdf]

Article DOI: https://doi.org/10.3201/eid.2703.203882

# Familial Clusters of Coronavirus Disease in 10 Prefectures, Japan, February−May 2020

## Appendix

**Appendix Table 1**. Websites of 10 prefectures tested for familial clusters of coronavirus disease, Japan, February−May, 2020*

| Prefecture | URL |
|---|---|
| Aomori | https://www.pref.aomori.lg.jp/welfare/health/wuhan-novel-coronavirus2020.html |
| Akita | https://www.pref.akita.lg.jp/pages/archive/47957 |
| Gunma | https://www.pref.gunma.jp/07/z87g_00016.html |
| Tochigi | http://www.pref.tochigi.lg.jp/e04/welfare/hoken-eisei/kansen/hp/coronakensahasseijyoukyou.html |
| Toyama | http://www.pref.toyama.jp/cms_sec/1205/kj00022166.html |
| Shiga | https://www.pref.shiga.lg.jp/ippan/kenkouiryouhukushi/yakuzi/310735.html |
| Okayama | https://www.pref.okayama.jp/page/667843.html |
| Kochi | https://www.pref.kochi.lg.jp/soshiki/130401/2020022900049.html |
| Saga | https://www.pref.saga.lg.jp/kiji00373220/index.html |
| Kagoshima | https://www.pref.kagoshima.jp/ae06/kenko-fukushi/kenko-iryo/kansen/kansensho/coronavirus.html |

*All websites were cited on July 22, 2020.

**Appendix Table 2.** Family transmission by asymptomatic case-patients in families 1–7 tested for familial clusters of coronavirus disease, Japan, February−May, 2020*

| Family | Cluster | Case-patient age, y/sex | Days since diagnosis of primary case –7 | –6 | –5 | –4 | –3 | –2 | –1 | 0 | 1 | 2 | 3 | 4 | 5 | 6 |
|---|---|---|---|---|---|---|---|---|---|---|---|---|---|---|---|---|
| 1 | School | <1/F | | | | | | | | × | | | | | | |
| | | 10/F | | | | | | | | | | | | | | |
| 2 | School | <1/M | | | | | | | | × | | | | | | |
| | | 40/M | | | | | | | | | | | | | | |
| | | 30/F | | | | | | | | | | | | | | |
| | | 80/M | | | | | | | | | | | | | | |
| | | 70/F | | | | | | | | | | | | | | |
| 3 | Hospital | 60/M | | | | | | | | × | | | | | | |
| | | 80/F | | | | | | | | | | | | | | |
| 4 | Hospital | 50/M | | | | | | | | × | | | | | | |
| | | 20/F | | | | | | | | | | | | | | |
| 5 | Nursing home | 80/F | | | | | | | | × | | | | | | |
| | | 60/M | | | | | | | | | | | | | | |
| 6 | Nursing home | 80/F | | | | | | | | × | | | | | | |
| | | 80/M | | | | | | | | | | | | | | |
| 7 | Restaurant | 60/M | | | | | | | | × | | | | | | |
| | | 60/F | | | | | | | | | | | | | | |
| | | 80/F | | | | | | | | | | | | | | |

*Blue, duration of symptoms; dark gray, case-patients were not included in nonfamilial clusters (e.g., school, hospital, occupational status); empty space, not applicable; orange, diagnosis; ×, primary case in a family. Age: <1 (<1–9 years); 10 (10–19 years), 20 (20–29 years), 30 (30–39 years), 40 (40–49 years), 50 (50–59 years), 60 (60–69 years), 70 (70–79 years), 80 (≥80 years). Primary asymptomatic cases in families 1 (<1/F), 2 (<1/M), 3 (60/M), 4 (50/M), 5 (80/F), 6 (80/F), and 7 (60/M) were diagnosed through outbreak investigation of other clusters, such as a school, nursing home, hospital, and restaurant.

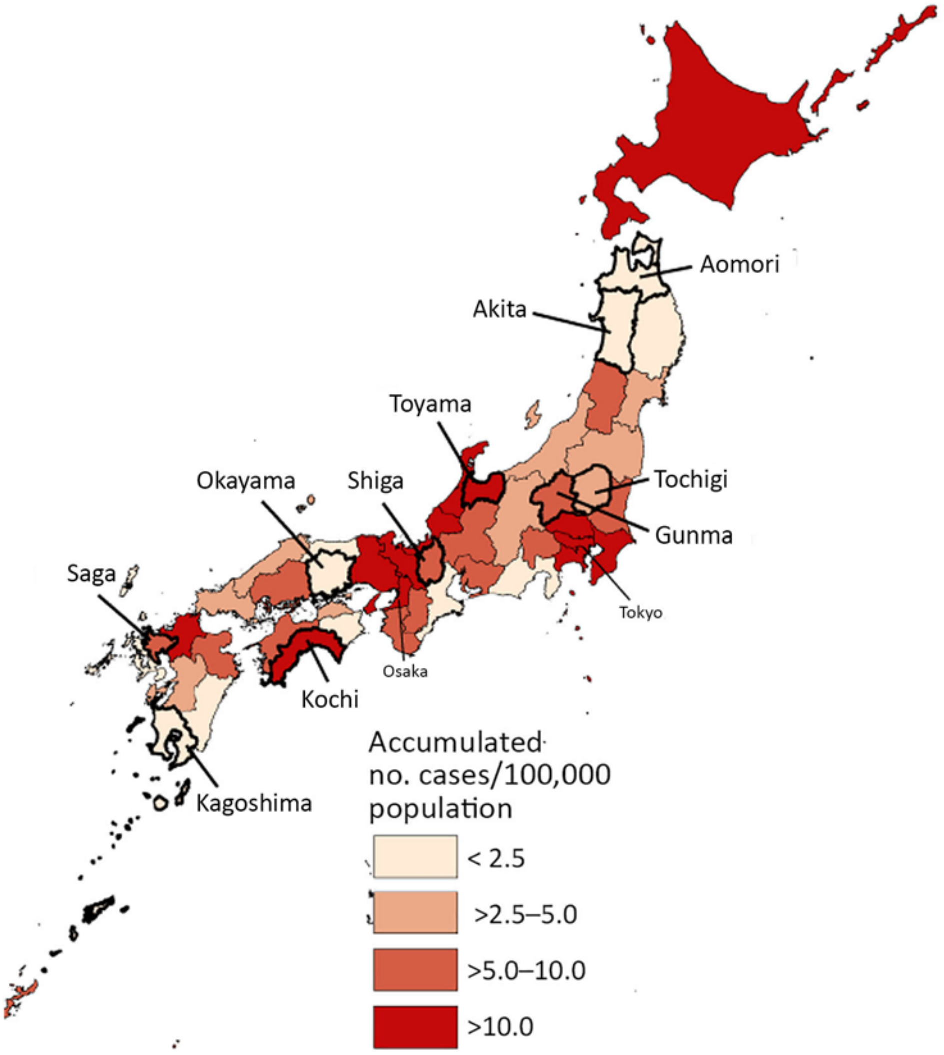


**Appendix Figure 1.** Locations of 10 prefectures tested for familial clusters of coronavirus disease and accumulated number of cases/100,000 population, Japan, February 22–May 31, 2020.

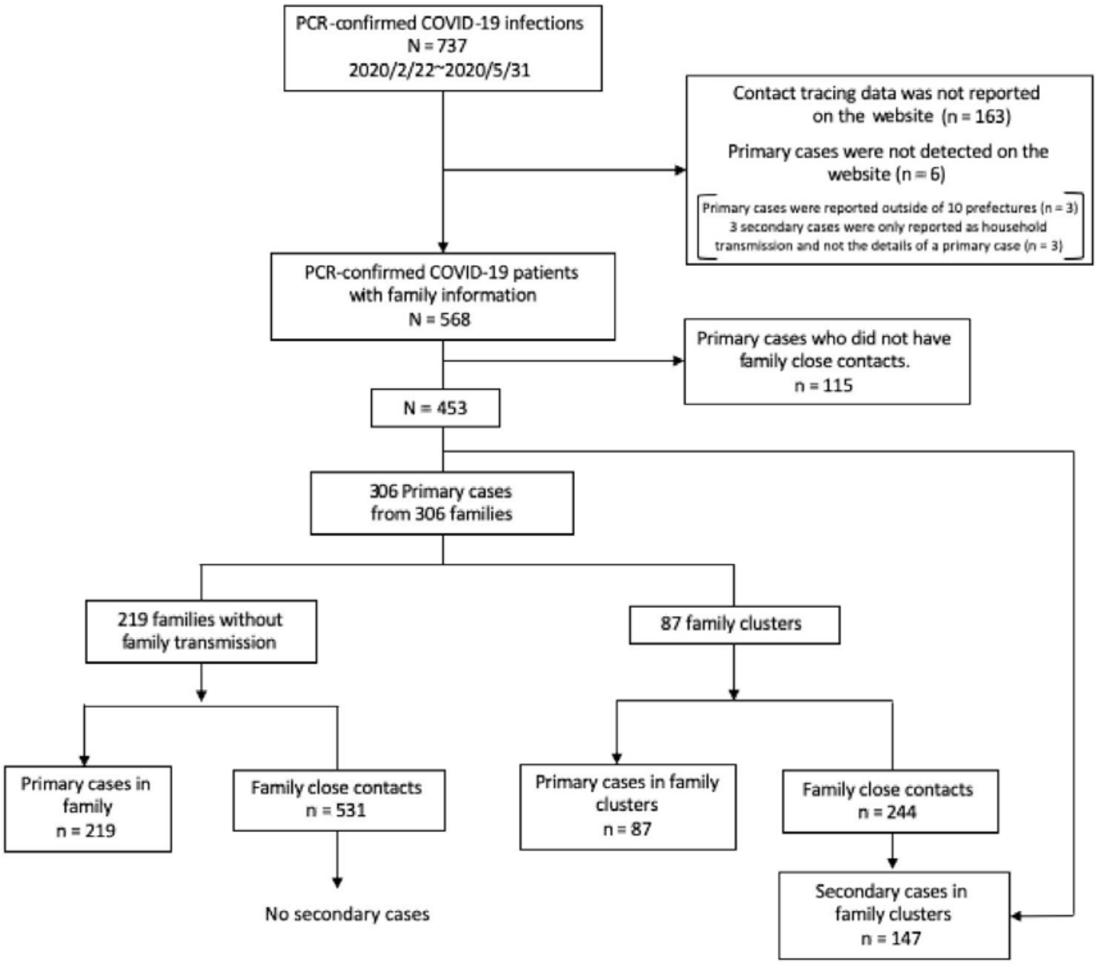


**Appendix Figure 2**. Study participants tested for familial clusters of coronavirus disease, Japan, February 22–May 31, 2020. COVID-19, coronavirus disease.
